# Pediatric Traumatic Brain Injury and Microvascular Blood-Brain Barrier Pathology

**DOI:** 10.1001/jamanetworkopen.2024.46767

**Published:** 2024-11-25

**Authors:** Josie L. Fullerton, Jennifer Hay, Charlotte Bryant-Craig, Josephine Atkinson, Douglas H. Smith, William Stewart

**Affiliations:** 1 School of Cardiovascular & Metabolic Health, University of Glasgow, Glasgow, United Kingdom; 2 The Francis Crick Institute, London, United Kingdom; 3 NHS Lanarkshire, Glasgow, United Kingdom; 4 Centre of Discovery Brain Sciences, University of Edinburgh, Edinburgh, United Kingdom; 5 Penn Centre for Brain Injury and Repair, Department of Neurosurgery, Perelman School of Medicine, University of Pennsylvania, Philadelphia; 6 School of Psychology & Neuroscience, University of Glasgow, Glasgow, UK; 7 Department of Neuropathology, Queen Elizabeth University Hospital, Glasgow, United Kingdom

## Abstract

**Importance:**

Pediatric traumatic brain injury (TBI) is a major cause of morbidity and mortality, with an increased risk of catastrophic outcome compared with adult TBI, including diffuse brain swelling and so-called second impact syndrome. Nevertheless, the biological substrates driving adverse outcomes in pediatric TBI remain poorly described.

**Objective:**

To compare neuropathological evidence of brain swelling and blood-brain barrier (BBB) disruption after moderate or severe acute TBI in adult vs pediatric case material.

**Design, Setting, and Participants:**

In this retrospective case series, cases of pediatric (aged 3-18 years) and adult (aged ≥19 years) TBI were accrued from January 1, 1979, to December 31, 2005, and underwent laboratory-based assessment of autopsy material from the Glasgow TBI Archive. Data analysis was performed from January 2019 to January 2024.

**Exposures:**

Single moderate or severe TBI.

**Main Outcomes and Measures:**

Evaluation of representative brain tissue sections stained for markers of endothelia (CD34) and BBB integrity (fibrinogen and immunoglobin G).

**Results:**

Eighty-one pediatric patients (mean [SD] age, 12.1 [4.6] years; 50 [62%] male) and 62 adult patients (mean [SD] age, 38.7 [12.9] years; 35 [56%] male) were studied. At autopsy, when present, brain swelling was more often diffuse and bilateral among pediatric patients (64 of 81 cases [83%]) when compared with adult patients (21 of 62 [34%]) (*P* < .001). Histologic evidence of BBB disruption was common in material from both adult (57 of 62 [91%]) and pediatric (65 of 81 [80%]) (*P* = .06) patients. In pediatric patients, however, this was a predominantly microvascular, capillary-level pathology, which was a less common finding in adult case material (mean [SD], 84.7% [8.6%] vs 31.2% [7.7%]; *P* < .001).

**Conclusions and Relevance:**

This autopsy case series of patients dying in the acute phase after single moderate or severe TBI provides neuropathological evidence of age-dependent differences in vascular pathology. Specifically, although BBB disruption in pediatric material was typically confined to microvascular, capillary-level vessels, in adult case material, BBB disruption more typically involved larger-diameter vessels. This observation of distinct microvascular pathology in pediatric acute TBI requires further investigation. In the meantime, this study presents an intriguing potential candidate pathology contributing to diffuse brain swelling in this age group.

## Key Points

**Question:**

What differences in vascular pathology exist among adult vs pediatric patients after single moderate or severe traumatic brain injury (TBI)?

**Findings:**

In this autopsy case series of individuals dying within 2 weeks of TBI, bilateral brain swelling was significantly more common among pediatric patients than among adults (83% vs 34%). Furthermore, although blood-brain barrier disruption was a largely capillary-level pathology among pediatric case material, this finding was significantly less frequent in adults (81% vs 31%).

**Meaning:**

These data suggest distinct microvascular pathology in pediatric patients following TBI, providing a potential substrate for diffuse brain swelling in this population.

## Teaser

This case series examines autopsy material from pediatric and adult patients dying within 2 weeks of traumatic brain injury to assess neuropathological evidence of brain swelling and blood-brain barrier disruption.

## Introduction

Traumatic brain injury (TBI) represents the leading cause of death and permanent disability in young children (aged <4 years) and adolescents (aged 15-18 years).^1,2^ In the US alone, pediatric TBI accounts for almost 500 000 emergency department visits and more than 7000 fatalities each year.^3,4^ In contrast to adults, pediatric patients appear especially vulnerable to catastrophic outcomes after TBI, in particular, diffuse brain swelling (DBS), which can arise after all injury severities, including mild TBI, where it is often colloquially referred to as second impact syndrome (SIS).^5,6^

Although first acknowledged in the 1950s, the association between DBS and pediatric TBI was not recognized until some years later.^7,8,9,10,11,12^ In rare and unpredictable instances, TBI of all severities, including mild TBI or concussion, leads to rapid-onset, severe neurologic impairment and, in many cases, death.^8^ It has been proposed that DBS may reflect TBI-induced vascular damage with associated disruption in cerebrovascular autoregulation, leading to uncontrolled brain swelling.^13,14^ Nevertheless, despite the catastrophic outcomes associated with DBS among pediatric patients, the underlying pathophysiologic drivers of this outcome have yet to be elucidated.

Evidence of widespread BBB disruption has been reported at autopsy in a high proportion of adults after a single moderate or severe TBI.^15^ Acute disruption in the BBB has also been reported in several animal models of TBI, including a clinically relevant swine model of concussion.^16^ Conceivably, such widespread vascular disturbance after injury may serve as a substrate for cerebral edema contributing to DBS. Furthermore, differences between adult and pediatric brains, including in BBB integrity, may render this population more susceptible to catastrophic outcome.^17,18,19^ However, thus far, no studies have explored the specific BBB response to TBI in pediatric patients.

We hypothesize that autopsy evidence of BBB disruption is common after TBI in material from pediatric patients. Furthermore, BBB disruption may have distinct features in this age group compared with adults, serving as possible substrates for diffuse DBS. To address these hypotheses, we examined autopsy material from pediatric and adult patients who died in the acute phase after single moderate or severe TBI for evidence of BBB disruption and, when present, the histologic characteristics of the involved vessels.

## Methods

### Study Design and Case Selection

For this autopsy case series, cases were obtained from the Glasgow TBI Archive of the Department of Neuropathology, Queen Elizabeth University Hospital, Glasgow, UK. Brain tissue samples were acquired after routine diagnostic autopsy between January 1, 1975, and December 31, 2006, with ethical approval and waiver of consent, in line with use of fully anonymized, autopsy derived tissue in research provided by the Greater Glasgow and Clyde Biorepository Governance Committee. The study followed the reporting guideline for case series.

Cases were randomly selected from the archive by a researcher (J.H.) independent of the pathology evaluation. Pediatric cases were selected from all available patients within the archive aged 3 to 18 years with a history of a single moderate or severe TBI who died in the acute phase after injury (≤2 weeks survival) and for which tissue blocks representing the anatomical regions of interest were available (n = 81). Adult cases comprised a randomly selected sample of the archived patients aged 19 to 60 years at death with the same injury exposure, survival time frame, and tissue availability (n = 62). For each case, available records of the original diagnostic autopsy examination were interrogated to provide information on degree and extent of TBI pathologies and any associated secondary pathologies. All autopsy procedures were conducted by experienced neuropathologists independent of this study using established, standardized approaches for neuropathologic examination of the brain at autopsy. Evidence in support of brain swelling included focal, symmetrical, or asymmetrical gyral compression, sulcal and/or ventricular effacement, or signs of internal herniation. Full demographic and clinical data for both study groups are provided in Table 1. Data on race and ethnicity are not available because these characteristics were not recorded at the time of original autopsy examinations.

**Table 1.** Demographic and Clinical Data for Adult and Pediatric Acute TBI Cohorts

Characteristic	No. (%) of cases of acute TBI^a^	
	Pediatric (n = 81)	Adult (n = 62)
Age, mean (SD) [range], y	12.1 (4.6) [3-18]	38.7 (12.9) [19-60]
Sex		
Male	50 (62)	35 (56)
Female	31 (38)	27 (44)
Postmortem delay, mean (SD) [range], h	47.6 (30.3) [3-120]	52.5 (40.4) [5-240]
Survival, mean (SD) [range], h	57.8 (56.9) [0-240]	71.7 (73.0) [0-264]
Cause of TBI		
Fall	12 (15)	27 (44)
Road traffic incident	59 (73)	23 (37)
Assault	8 (10)	11 (18)
Unknown	2 (2)	1 (2)
Cause of death		
Head injury	73 (90)	52 (84)
Bronchopneumonia	0	2 (3)
Heart disease	1 (1)	0
Multiple injuries	4 (5)	8 (13)
Unknown	3 (4)	0

Abbreviation: TBI, traumatic brain injury.

^a^ Unless otherwise indicated.

### Tissue Preparation

At the time of the original diagnostic autopsy, whole brains were immersion fixed in 10% formal saline for a minimum of 3 weeks. Once dissected, brains were sampled following a standardized block selection protocol and processed to paraffin tissue blocks as previously described.^20^ For this study, blocks were selected from a coronal slice of the cerebral hemispheres at the level of the lateral geniculate body and remote from any focal TBI pathology to include the following: the cingulate gyrus with adjacent corpus callosum; the superior frontal sulcus and adjacent middle frontal gyrus; the insular cortex; the hippocampal formation, entorhinal cortex, and adjacent medial temporal lobe; and the thalamus with adjacent internal capsule.

### Immunohistochemistry

From each tissue block, 8-μm sections were cut using a rotary microtome (Leica Microsystems) and mounted onto Superfrost Plus microscope slides (Cellpath). Sections were deparaffinized and rehydrated before incubation in 3% aqueous hydrogen peroxide for 15 minutes to quench endogenous peroxidase activity. Antigen retrieval was performed using a microwave pressure cooker in preheated 0.1-mmol/L Tris EDTA buffer (pH 8) for 8 minutes. Sections were blocked for 30 minutes using normal horse serum (Vector Laboratories) in Optimax buffer (BioGenex). Once blocked, sections were incubated for 20 hours at 4 °C with 1 of the following polyclonal rabbit antihuman primary antibodies: fibrinogen (1:17 500; Agilent Technologies), IgG (1:8000; Dako), or CD34 (1:100; Leica Biosystems). After overnight incubation, sections were rinsed and processed with Vector Labs Elite Universal ABC kit (Vector Laboratories) per the manufacturer’s instructions. Sections were incubated for 30 minutes at room temperature with a biotinylated universal secondary antibody, followed by avidin-biotin complex (1:50) incubation for 30 minutes. Finally, visualization was achieved using 3, 3′-diaminobenzidine peroxidase substrate kit (Vector Laboratories), and sections were counterstained with hematoxylin, rinsed and dehydrated, and then coverslipped using clear mounting medium (Sigma Aldrich). In parallel with test sections, known positive tissue and sections with primary antibody omitted were processed as standard controls for antibody specificity (for all 3 antibodies in all experimental runs).

### Whole Slide Scanning and Immunohistochemical Analysis

After staining, sections were viewed using a Leica DMRB light microscope (Leica Microsystems) and digitized by scanning at ×20 using a Hamamatsu Nanozoomer 2.0-HT slide scanner, with the resultant images viewed via the SlidePath Digital Image Hub application (Leica Microsystems). All observations were conducted by 2 independent observers (J.L.F. and J.A.) who were blind to demographic and clinical data. Each tissue section was reviewed for the extent and distribution of fibrinogen or IgG immunoreactivity using established semiquantitative techniques.^15^ To assess fibrinogen and IgG immunoreactivity, we semiquantitatively assessed 4 areas of interest: the cingulate gyrus and cingulate sulcus (including the medial and lateral corpus callosum), hippocampus, insular cortex, and thalamus (including the internal capsule of the thalamus). The frequency and extent of fibrinogen or IgG immunoreactivity were determined as absent (0), sparse (1), moderate (2), or extensive (3). Representative examples of immunohistochemical findings and corresponding semiquantitative scores are shown in eFigure 1 in Supplement 1, using previously published methods of semiquantitative analyses.^15^ Interrater reliability for semiquantitative scoring was 98% (Cohen κ = 0.96), with discrepant scores reviewed and a final, consensus score agreed between observers. For the assessments of vessel diameter, adjacent sections from the cingulate gyrus where abnormal, moderate, or extensive perivascular fibrinogen immunoreactivity (score 2 or 3) was observed in any layer of the cingulate gyrus were used. These sections were subject to blinded, unbiased assessment of the diameter of all vessels (CD34-stained sections) and of vessels showing BBB disruption (fibrinogen-stained sections). To achieve this, a 10 × 10 grid composed of 300 × 300-μm squares was placed over the neocortical gray matter. Within this grid, vessels transecting the left or lower gridlines in each counting square were highlighted on the image and the minimum cross-sectional diameter for that vessel recorded. Vessels landing wholly within or transecting the upper or right gridlines of a counting square were not counted.

### Statistical Analysis

Data were analyzed using GraphPad Prism, version 10.0 (GraphPad Software). To assess differences between groups, the Fisher exact or 2-tailed, unpaired *t* test was performed as appropriate, with statistical significance set at at 2-sided *P* < .05. Data analysis was performed from January 2019 to January 2024.

## Results

Eighty-one pediatric patients (mean [SD] age, 12.1 [4.6] years; 50 [62%] male and 31 [38%] female) and 62 adult patients (mean [SD] age, 38.7 [12.9] years; 35 [56%] male and 27 [44%] female) were studied. Causes of death, survival time (pediatric patients: mean [SD], 57.8 [56.9] hours; range, 0-240 hours; adult patients: mean [SD], 71.7 [73] hours; range, 0-264 hours), and postmortem intervals (pediatric patients: mean [SD], 47.6 [30.3] hours; range, 3-120 hours; adult patients: mean [SD], 52.5 [40.4] hours; range, 5-240 hours) were comparable in the groups (Table 1). Among pediatric cases, the most common mechanism of injury was road traffic incidents, whereas among adults, the most common cause of TBI was falls.

### Bilateral Brain Swelling in Acute Pediatric TBI

At autopsy, macroscopic evidence of brain swelling was more prevalent among pediatric (67 of 81 cases [83%]) than adult (40 of 62 cases [65%]) (*P* = .02, Fisher exact test) acute TBI cases (Table 2). Furthermore, when brain swelling was present, this swelling was typically bilateral in pediatric TBI cases (64 of 81 [83%]), which was a considerably more frequent finding than among adult cases (21 of 62 [34%]) (*P* < .001). Consistent with the degree of brain swelling, histologic evidence of ischemic brain injury was more frequent among pediatric case material (67 of 81 [83%]) than among adult case material (40 of 62 [65%]) (*P* = .02).

**Table 2.** Neuropathology Findings at Autopsy

Autopsy neuropathologic observation	No. (%) of cases of acute TBI		*P* value^a^
	Pediatric (n = 81)	Adult (n = 62)	
Macroscopic findings			
Skull fracture	58 (71)	37 (59)	.16
Raised intracranial pressure	67 (83)	45 (73)	.16
Brain swelling	67 (83)	40 (65)	.02
Bilateral brain swelling	64 (83)	21 (34)	<.001
Extradural hematoma	11 (14)	10 (18)	.81
Subdural hematoma	33 (43)	24 (39)	.86
Subarachnoid hemorrhage	40 (50)	21 (34)	.09
Intracerebral hemorrhage	31 (38)	33 (54)	.09
Contusion	71 (88)	50 (81)	.35
Microscopic findings			
Diffuse axonal injury	39 (48)	22 (35)	.17
Ischemic brain injury	67 (83)	40 (65)	.02

Abbreviation: TBI, traumatic brain injury.

^a^ Fisher exact text.

### Evidence of Widespread Acute BBB Disruption After TBI in Pediatric and Adult Patients

**Figure 1.**
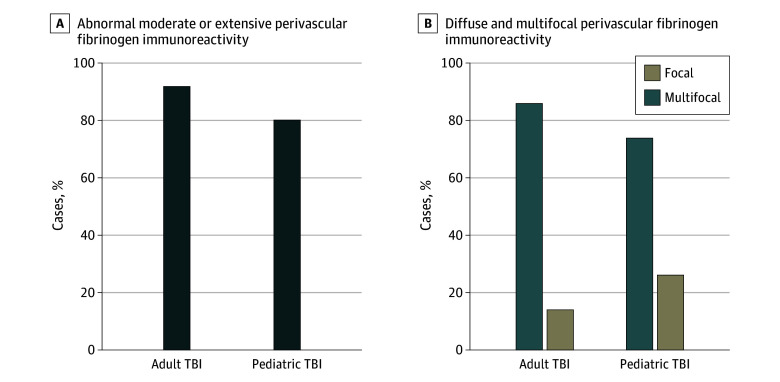
Abnormal Fibrinogen Immunoreactivity After Acute Traumatic Brain Injury (TBI) in Adult and Pediatric Patients A, Moderate to extensive perivascular fibrinogen immunoreactivity (score, 2-3) was observed in a similar proportion of both adult and pediatric material after acute TBI. B, Typically, this was diffuse and multifocal, involving 2 or more anatomical regions in most of the adult and pediatric material.

Abnormal moderate or extensive perivascular fibrinogen immunoreactivity was present in a similar proportion of pediatric (65 of 81 [80%]) and adult (57 of 62 [91%]) (*P* = .06) cases (Figure 1A). Typically, this abnormal fibrinogen immunoreactivity was observed as multifocal, diffuse immunoreactive profiles across 2 or more regions in pediatric (48 of 65 [74%]) and adult (49 of 57 [86%]) (*P* = .12) cases (Figure 1B). These appearances were corroborated in sections stained for IgG in which abnormal, multifocal, perivascular immunoreactivity was observed in 64 of 81 pediatric cases (79%) and 53 of 62 adult cases (86%) (*P* = .26) (eFigure 2 in Supplement 1).

### BBB Disruption After Acute Pediatric TBI

**Figure 2.**
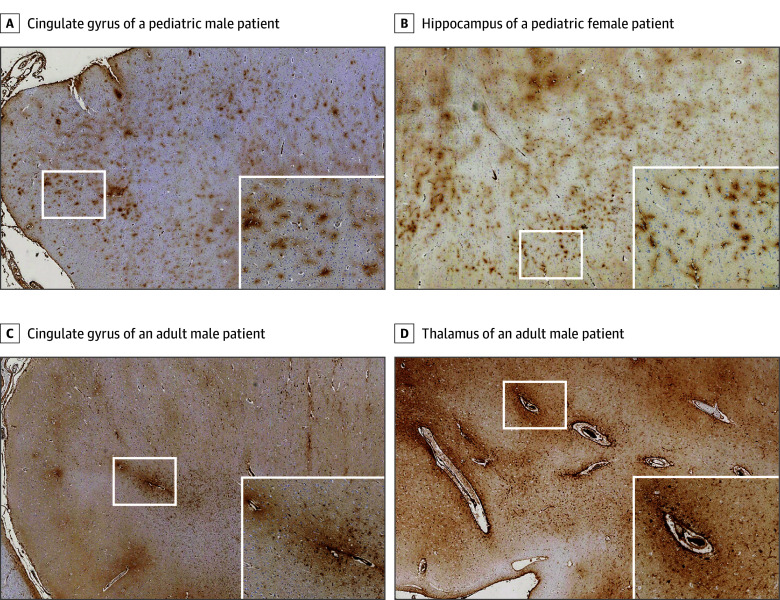
Representative Fibrinogen Immunoreactivity in Adult and Pediatric Acute Traumatic Brain Injury (TBI) In pediatric TBI material, abnormal fibrinogen staining appeared as numerous, punctate foci of immunoreactivity surrounding small intraparenchymal vessels (white squares), as shown in the cingulate gyrus of pediatric male patient who died 72 hours after an assault (A) and the hippocampus of a pediatric female patient who died 24 hours after an assault (B). In contrast, adult patients with TBI displayed more confluent areas of abnormal fibrinogen staining around medium to larger vessels, with little to no punctate pattern staining (white squares), as shown in the cingulate gyrus (C) and thalamus (D) of an adult male patient surviving 8 days after a fall (original magnification ×12.5; inset: original magnification ×50).

**Figure 3.**
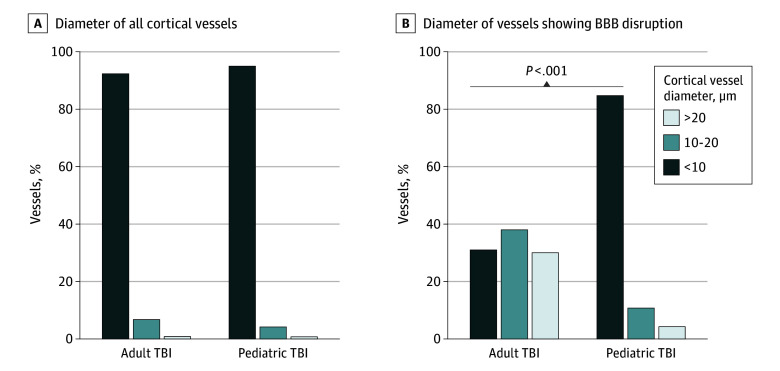
Diameter of All Cortical Vessels in the Cingulate Gyrus of Adult and Pediatric Traumatic Brain Injury (TBI) Compared With the Diameter of Disrupted Vessels A, In the cingulate gyrus, the diameter of cortical vessels in sections stained for CD34 was typically 10 μm or less in both adult and pediatric case material. B, Nevertheless, although in pediatric case material most vessels demonstrating abnormal fibrinogen staining were 10 μm or less in diameter, this was a far less frequent observation in adult case material. BBB indicates blood-brain barrier.

Although the extent and distribution of BBB disruption after moderate or severe acute TBI were similar in adult and pediatric material, the pattern of vascular involvement was disparate. Thus, although in pediatric TBI material, moderate or extensive abnormal fibrinogen immunoreactivity was characterized by numerous punctate, perivascular foci of staining surrounding small-diameter intraparenchymal vessels (Figure 2A and B; eFigure 3A-C in Supplement 1), adult material more typically showed fewer and larger confluent immunoreactive foci of staining, which appeared localized to medium to larger-diameter vessels (Figure 2C and D; eFigure 3D-F in Supplement 1). Consistent with this observation, although in material stained for CD34 a similar proportion of cortical vessels in adult and pediatric material was 10 μm or less in diameter (Figure 3A), evidence of abnormal fibrinogen immunoreactivity appeared disproportionately distributed to these smallest-diameter vessels in pediatric TBI sections. Specifically, in pediatric TBI material, although a mean (SD) of 84.7% (8.6%) of vessels in each case demonstrated abnormal fibrinogen staining of 10 μm or less in diameter, only 31.2% (7.7%) of vessels in adults had abnormal fibrinogen staining of 10 μm or less in diameter (*P* < .001, *t* test) (Figure 3B). Indeed, preferential microvascular BBB disruption with a less than 10-μm diameter was present in only 1 adult case, an individual aged 20 years, who also showed evidence of unilateral brain swelling.

## Discussion

In this case series, we demonstrate autopsy evidence of DBS and BBB disruption in examination of autopsy material from adult and pediatric patients after a single moderate or severe acute TBI. Notably, although DBS was documented in most pediatric cases and was typically bilateral, such bilateral swelling was a far less common observation after adult TBI. Furthermore, although BBB disruption was widespread across both cohorts, there was a clear difference in the vascular bed involved in pediatric vs adult material. Specifically, although in pediatric material BBB disruption appeared as a pathology predominantly associated with capillary-sized vessels, in adult material this microvascular vulnerability was considerably less common, with medium to larger vessels more typically involved. Combined, these data support our hypothesis that there is pathologic disparity in BBB disruption between pediatric and adult patients dying after single moderate or severe acute TBI.

Our observation of autopsy evidence of DBS in a much higher proportion of pediatric compared with adult patients after single moderate or severe acute TBI is consistent with prior clinical and autopsy studies.^21^ Furthermore, in clinical studies, this widespread DBS has been associated with high mortality among the pediatric population.^8,21^ Evidence suggests that DBS may arise due to a combination of increased intracerebral blood, either as an increase in cerebral blood volume or as a redistribution of intracranial blood from vessels located in the pia to intraparenchymal vessels and increased brain volume due to edema.^10,22,23^ Our finding of widespread BBB disruption in both adult and pediatric material would provide a potential substrate for vasogenic edema contributing to DBS in this population.^24,25^

Despite DBS being considerably more common among pediatric cases, the prevalence of BBB disruption was similar in both pediatric and adult case material. In keeping with previous work, in adult case material, histologic evidence of BBB disruption was present as fibrinogen immunoreactivity around small to medium vessels and extending into the surrounding brain parenchyma.^15^ Typically, these vessels were 10 μm in diameter or greater in adults, consistent with vessels of arteriole or venule size and larger. However, in pediatric case material, the vessels involved were overwhelmingly capillary level (ie, <10 μm in diameter). This striking and distinctive microvascular BBB disruption associated with pediatric TBI might, at least in part, contribute to the more catastrophic association between BBB and DBS in this population.

In keeping with the autopsy nature of this study, all injuries were moderate or severe in severity. However, among pediatric patients, DBS may follow even after mild TBI or concussion.^26,27^ In such circumstances, there is often, although not always, a history of recent mild TBI in the days before presentation, leading to its description as SIS,^8^ although this association might simply be reflective of recall bias in largely retrospective case history ascertainment rather than evidence of a requirement for 2 injuries in short succession. Thankfully, such cases are thought to be rare, with a previous review identifying only 43 reported instances in the published literature,^28^ and 1 additional case reported since.^29^ Notably, SIS is almost exclusively reported in adolescent or young adult patients, with a mean age of published cases of 17.8 years (range, 10-29 years). In this context, it is of note that only 1 adult in this study, aged 20 years, showed capillary-level microvascular BBB disruption typically seen in our pediatric material. This finding presents the intriguing possibility that the distinct capillary-level BBB disruption seen here might contribute to the catastrophic presentation of SIS. Additional work to explore this prospect is required, including formal assessment of BBB permeability at autopsy in cases of SIS, where opportunity presents.

### Limitations

This study has some limitations. The study used postmortem patient samples from the Glasgow TBI Archive, and although this archive is comprehensive, it is primarily representative of a Scottish White population. Studies in wider populations are required. Furthermore, the retrospective nature of case ascertainment is such that there are limitations on available clinical information, including a lack of access to diagnostic imaging studies, which would provide opportunity to review potential imaging correlates of the observations reported herein. There have been many advances in acute TBI care in recent decades. As such, the largely historical nature of case acquisition may not be reflective of current patient profiles in clinical settings. There is, therefore, a clear need to consider the implications of our data in context of current patient management, in particular, the presentation of DBS in pediatric TBI populations.

## Conclusions

In this autopsy case series examining material from individuals dying in the acute phase following single moderate or severe TBI, we report striking differences in vascular pathology between adult and pediatric patients. Specifically, although evidence of BBB disruption in acute TBI was typically confined to microvascular, capillary-level vessels in pediatric cases, in adult case material, larger-diameter vessel involvement was the norm. This observation of distinctive vascular pathology in pediatric acute TBI requires further investigation; however, in the meantime, it presents an intriguing potential candidate pathology contributing to DBS and SIS in this age group.

## Notes

Supplement 1. **eFigure 1.** Representative Examples of Fibrinogen (FBG) Semi-Quantitative Scoring **eFigure 2.** Abnormal Immunoglobulin G (IgG) Immunoreactivity Following Acute TBI in Adult and Pediatric Patients **eFigure 3.** Representative Fibrinogen (FBG) Immunoreactivity in Adult and Pediatric Acute TBI

Supplement 2.
Data Sharing Statement
