# Supplementary material for: Pediatric Traumatic Brain Injury and Microvascular Blood-Brain Barrier Pathology
Source: JAMA Netw Open. 2024 Nov 25;7(11):e2446767. doi: 10.1001/jamanetworkopen.2024.46767 (PMC11589795; doi:10.1001/jamanetworkopen.2024.46767)
Supplement: Supplement 1. — eFigure 1. Representative Examples of Fibrinogen (FBG) Semi-Quantitative Scoring eFigure 2. Abnormal Immunoglobulin G (IgG) Immunoreactivity Following Acute TBI in Adult and Pediatric Patients eFigure 3. Representative Fibrinogen (FBG) Immunoreactivity in Adult and Pediatric Acute TBI [file jamanetwopen-e2446767-s001.pdf]

Supplementary Online Content

Fullerton JL, Hay J, Bryant-Craig C, Atkinson J, Smith DH, Stewart W. Pediatric traumatic brain injury and microvascular blood-brain barrier pathology. *JAMA Netw Open*. 2024;7(11):e2446767. doi:10.1001/jamanetworkopen.2024.46767

eFigure 1. Representative Examples of Fibrinogen (FBG) Semi-Quantitative Scoring

eFigure 2. Abnormal Immunoglobulin G (IgG) Immunoreactivity Following Acute TBI in Adult and Pediatric Patients

eFigure 3. Representative Fibrinogen (FBG) Immunoreactivity in Adult and Pediatric Acute TBI

This supplementary material has been provided by the authors to give readers additional information about their work.

© 2024 Fullerton JL et al. *JAMA Network Open.*

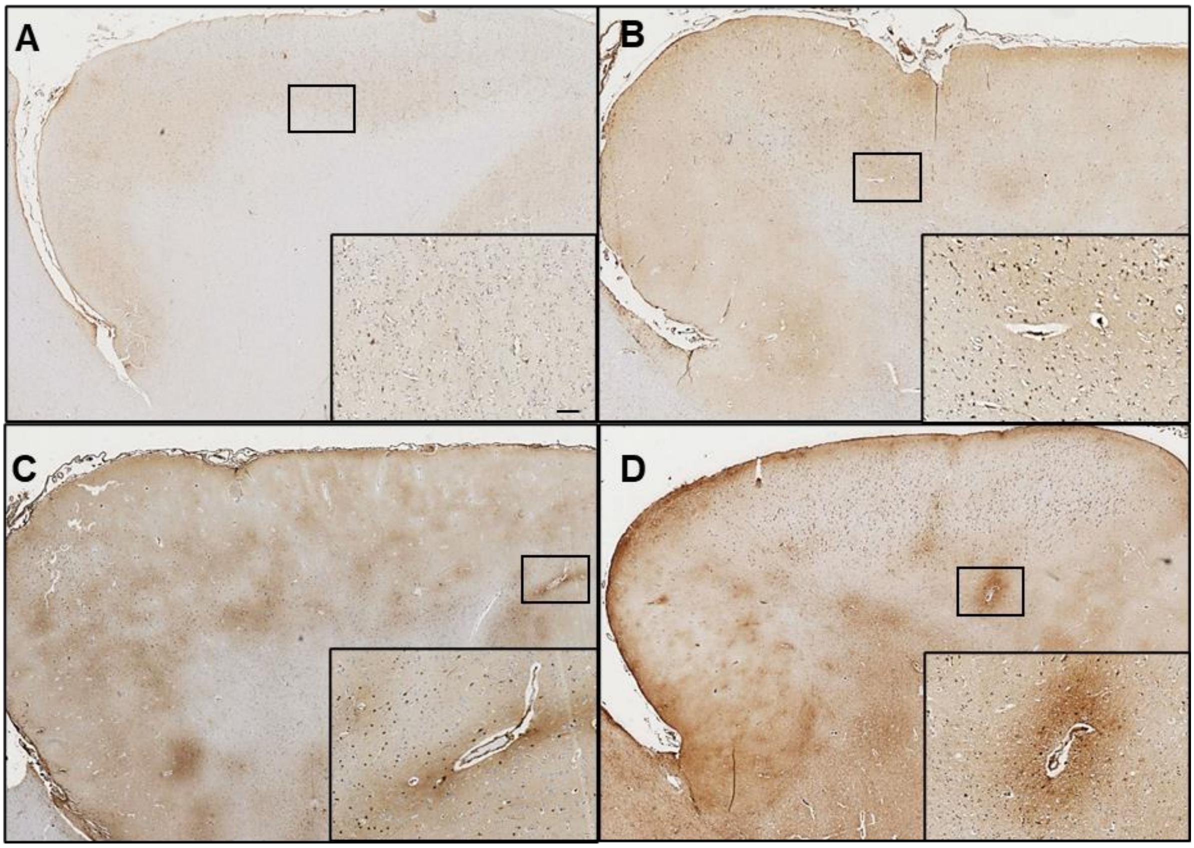

eFigure 1. Representative examples of fibrinogen (FBG) semi-quantitative scoring. (A) Section from the superior frontal gyrus of a 13-year-old female who survived three days after a road traffic accident; section shows no abnormal FBG immunoreactivity (score of 0). **(B)** Limited sparse perivascular FBG immunoreactivity in the same region of a 49-year-old male who died 48 hours after a fall (score of 1). **(C)** Diffuse, moderate perivascular FBG staining in a 40-year-old female 10 days after an assault (score of 2). **(D)** More extensive and widespread abnormal perivascular FBG immunoreactivity in the superior frontal gyrus of an 18-year-old male who survived three days after an assault (score of 3). Scale bar = 100 µm.

© 2024 Fullerton JL et al. *JAMA Network Open.*

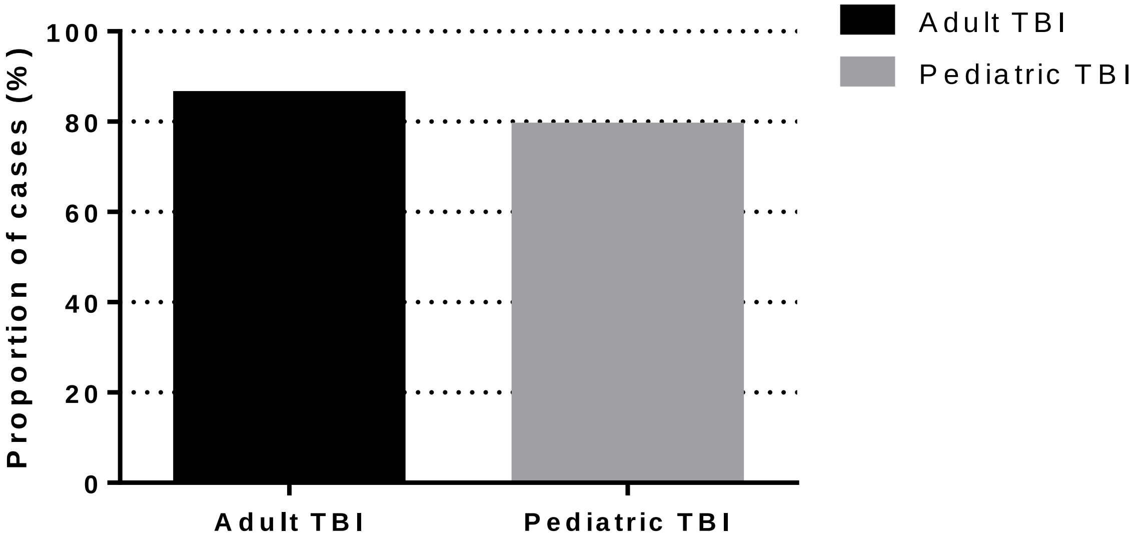

eFigure 2: Abnormal Immunoglobulin G (IgG) immunoreactivity following acute TBI in adult and pediatric patients. Moderate to extensive perivascular IgG immunoreactivity (score 2-3) was observed in adult (86%) and pediatric (79%) material following acute TBI (*P*=.26; Fisher's exact test).

© 2024 Fullerton JL et al. *JAMA Network Open.*

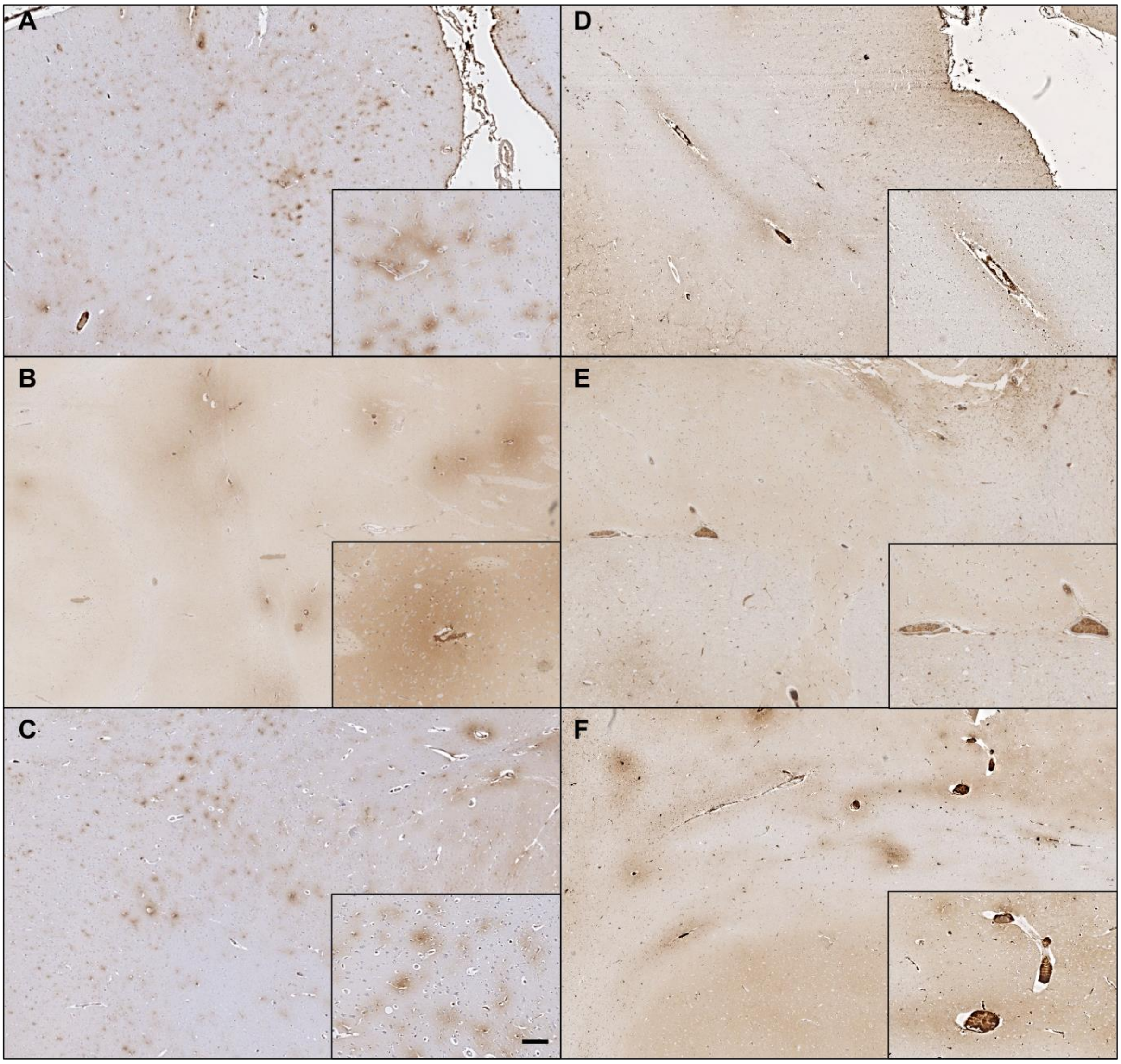

eFigure 3: Representative fibrinogen (FBG) immunoreactivity in adult and pediatric acute TBI. In pediatric TBI material, abnormal FBG immunoreactivity was displayed as numerous, punctate foci surrounding small intra-parenchymal vessels, as shown in the (A) insular cortex, (B) thalamus and (C) hippocampus and of an 11-year-old female, who died 5 days after a fall. In contrast, adult TBI patients displayed more confluent areas of abnormal FBG staining around medium to large vessels, with little to none of the more punctate distribution seen in pediatric TBI material, as shown in the (D) insular cortex, (E) thalamus and (F) hippocampus of a 31-year-old male who all died 29 hours after a road traffic accident. Scale bar 100µm.

© 2024 Fullerton JL et al. *JAMA Network Open.*
