# Supplementary material for: Pediatric Traumatic Brain Injury and Microvascular Blood-Brain Barrier Pathology
Source: JAMA Netw Open. 2024 Nov 25;7(11):e2446767. doi: 10.1001/jamanetworkopen.2024.46767 (PMC11589795; doi:10.1001/jamanetworkopen.2024.46767)
Supplement: Supplement 2. — Data Sharing Statement [file jamanetwopen-e2446767-s002.pdf]

Data Sharing Statement

Fullerton. Pediatric Traumatic Brain Injury and Microvascular Blood-Brain Barrier Pathology. *JAMA Netw Open*. Published November 25, 2024. doi:10.1001/jamanetworkopen.2024.46767

Data
Data available: No

Additional Information
Explanation for why data not available: All the data related to the observations are presented in the article text and figures. The photomicrographs used to generate all the findings across brain regions along with the quantification are available upon reasonable request.
